# Supplementary material for: Placental lesions in stillbirths: A case-control study using the Amsterdam criteria and predictive models at a UK tertiary unit
Source: PLoS One. 2025 Dec 26;20(12):e0338592. doi: 10.1371/journal.pone.0338592 (PMC12742796; doi:10.1371/journal.pone.0338592)
Supplement: Supplementary file 1 [file pone.0338592.s001.odt]

Table S1. Case-control characteristics for singleton term pregnancies

| Maternal Characteristics | Stillbirth n=28 | Livebirth n=28 | p-value |
| --- | --- | --- | --- |
| Age, year | 29.8 (±5.4) (n=28) | 29.4 (±4) (n=28) | N/S |
| BMI kg/m2 | 24.4 (21.2-32.8) (n=27) | 25.7 (23.2-29.7) (n=28) | N/S |
| Parity | 1 (0.0-3.7) (n=28) | (0.0-1.0) (n=28) | 0.01 |
| Ethnicity (n, %) White Black Asian Mixed Other | (n=25) 16 (64.0%) 3 (12.0%) 5 (20.0%)  0 (0.0%) 1 (4.0%) | (n=28) 26 (92.9%) 1 (3.6%) 1 (3.6%) (0.0%) 0.0 (0.0%) | N/S |
| Cigarette smoker (n,%) | 4 (14.3%) (n=28) | 1 (3.6%) (n=28) | N/S |
| Substance abuse (n, %) | 3 (11.5%) (n=26) | 0 (0.0%) (n=18) | N/S |
| Deprivation Index (n, %) 1-3 (most deprived areas) 4-6 7-10 (least deprived areas) | (n = 27) 18 (66.7%) 3 (11.1%) 6 (22.2%) | (n=27) 13 (48.1%) 3 (11.1%) 11 (40.8%) | 0.01 |
| Conception Type (n, %) Spontaneous | (n=25) 25 (100.0%) | (n=28) 28 (100.0%) | N/S |
| Mean arterial blood pressure at booking | 85.6 (±8.2) (n=24) | 86.9 (±7.3) (n=28) | N/S |
| Previous stillbirth (n,%) | 1 (3.7%) (n=27) | 1 (3.6%) (n=28) | N/S |
| Gestational age at delivery (weeks) | 39.9 (±1.2) (n=28) | 39.7 (±1.4) (n=28) | N/S |
| Fetal weight centile adjusted by GA using the Intergrowth calculator (31) | 30.4 (8.6-53.2) (n=27) | 72.2 (53.1-88.5) (n=27) | <0.001 |
| Fetal weight centile < 10th centile (31) | 8 (29.6%) (n=27) | 1 (3.7%) (n=27) | 0.01 |

For continuous variables data is expressed as either mean ±SD or median and interquartile range (25th-75th). Discrete variables are presented as raw number and percentage. Significant p-value < 0.05. ART: assisted reproductive technology, BMI: body mass index, N/S: not significance
