# Supplementary material for: Placental lesions in stillbirths: A case-control study using the Amsterdam criteria and predictive models at a UK tertiary unit
Source: PLoS One. 2025 Dec 26;20(12):e0338592. doi: 10.1371/journal.pone.0338592 (PMC12742796; doi:10.1371/journal.pone.0338592)
Supplement: Supplementary file 2 [file pone.0338592.s002.odt]

*

*

*

*

Figure S1. The frequency of placental lesions either in isolation or combined observed in singleton stillbirths (n=28) and livebirths (n=28) delivered at term.

*Statistically significant (p<0.05), FIR: fetal inflammatory response, DVM: delayed villous maturation, FMV: fetal vascular malperfusion, MVM: maternal vascular malperfusion, MIR: maternal inflammatory response, VUE: villitis of unknown etiology
