# Supplementary material for: Placental lesions in stillbirths: A case-control study using the Amsterdam criteria and predictive models at a UK tertiary unit
Source: PLoS One. 2025 Dec 26;20(12):e0338592. doi: 10.1371/journal.pone.0338592 (PMC12742796; doi:10.1371/journal.pone.0338592)
Supplement: Supplementary file 3 [file pone.0338592.s003.odt]

a)

A screenshot of a data

Description automatically generated

b)

A screenshot of a graph

Description automatically generated

c)

A screenshot of a graph

Description automatically generatedA screenshot of a graph

Description automatically generated

e)

d)

A screenshot of a graph

Description automatically generatedA graph with numbers and lines

Description automatically generated with medium confidence

Figure S2. Forest plot demonstrating the odds ratio of (a) MVM, (b) FVM, (c) MIR and FIR, (d) VUE and (e) DVM in stillbirth and livebirth placentas. 

DVM: delayed villous maturation, FMV: fetal vascular malperfusion, FIR: fetal inflammatory response, MIR: maternal inflammatory response, MVM: maternal vascular malperfusion, VUE: villitis of unknown etiology
