# Supplementary material for: Placental lesions in stillbirths: A case-control study using the Amsterdam criteria and predictive models at a UK tertiary unit
Source: PLoS One. 2025 Dec 26;20(12):e0338592. doi: 10.1371/journal.pone.0338592 (PMC12742796; doi:10.1371/journal.pone.0338592)
Supplement: Supplementary file 4 [file pone.0338592.s004.odt]

Table S2. Clinical performance of PET and gestational hypertension risk assessment in the stillbirth cohort.

|  | Sensitivity % (95%CI) | Specificity % (95%CI) | Positive predictive value % (95%CI) | Negative predictive value % (95%CI) | Area under the curve % (95%CI) | p-value |
| --- | --- | --- | --- | --- | --- | --- |
| NICE criteria | 12.5 (0.3-52.7) | 89.6 (78.8-95.5) | 12.5 (2.0-50.4) | 89.1 (86.1-91.5) | 0.51 (0.39-0.63) | 0.9 |
| FMF 1st trimester algorithm# | 75.0 (34.9-96.8) | 64.1 (51.1-75.7) | 20.7 (13.5-30.4) | 95.3 (85.9-98.6) | 0.76 (0.65-0.86) | 0.002* |

#Calculated using a cut-off of 1/300 (≥0.003), *significant p<0.05

CI: confidence interval, FMF: Fetal Medicine Foundation, NICE: National Institute for Health and Care Excellence
