# Supplementary material for: Placental lesions in stillbirths: A case-control study using the Amsterdam criteria and predictive models at a UK tertiary unit
Source: PLoS One. 2025 Dec 26;20(12):e0338592. doi: 10.1371/journal.pone.0338592 (PMC12742796; doi:10.1371/journal.pone.0338592)
Supplement: Supplementary file 5 [file pone.0338592.s005.odt]

Table S3. Clinical performance of MVM risk assessment in the stillbirth cohort using the FMF algorithm embedded in the Tommy’s app

|  | Sensitivity % (95%CI) | Specificity % (95%CI) | Positive predictive value % (95%CI) | Negative predictive value % (95%CI) | Area under the curve % (95%CI) | p-value |
| --- | --- | --- | --- | --- | --- | --- |
| All MVM combined | 54.2 (32.8-74.4) | 66.7 (51.6-79.6) | 44.8 (32.1-58.3) | 74.4 (64.3-82.4) | 0.63 (0.51-0.74) | 0.07 |
| Global/partial MVM lesions | 63.6 (30.8-89.1) | 63.9 (50.6-75.8) | 24.3 (17.9-32.2) | 94.3 (82.2-98.3) | 0.70 (0.58-0.8) | 0.02* |
| Segmental/ complete MVM lesions | 53.8 (25.1-80.8) | 62.7 (49.1-75.0) | 24.1 (14.8-36.8) | 86.0 (76.9-92.0) | 0.58 (0.45-0.69) | 0.4 |

#Calculated using a cut-off of 1/300 (≥0.003), *significant p<0.05

CI: confidence interval, FMF: Fetal Medicine Foundation, MVM: maternal vascular malperfusion
